# Octreotide Infusion for the Treatment of Congenital Chylothorax

**DOI:** 10.1155/2020/8890860

**Published:** 2020-11-11

**Authors:** Sumayyah Ahmed Nezar Kobeisy, Abdulaziz Alkhotani, Mohammed M. Barzanji

**Affiliations:** ^1^Dr. Soliman Fakeeh Hospital, Jeddah, Saudi Arabia; ^2^Umm Al-Qura University, Mecca, Saudi Arabia

## Abstract

Congenital chylothorax is an uncommon cause of pleural effusion in the pediatric age group, and it should be kept in consideration when evaluating a neonate with pleural effusion, as it is the commonest cause of pleural effusion in this age group (Tutor, 2014). No commonly accepted guidelines have been published so far regarding the management of congenital chylothorax in the neonate, but trials of octreotide have appeared to be promising (Bellini et al., 2018). We present a neonate with congenital chylothorax successfully treated with octreotide infusion.

## 1. Introduction

Idiopathic congenital chylothorax is a rare condition that results from the abnormal accumulation of the lymphocyte predominant chyle in the pleural space and is the most common cause of pleural effusion in the perinatal period [1]. It is approximated to affect 1 in 10,000–15,000 births with mortality rates ranging between to 20–60%. The wide variance in reported mortality rates can be attributed to maturational age of the newborn, severity of the pleural effusion, and other associated factors [2]. Despite this, no guidelines have been unanimously established in the treatment and management of congenital chylothorax [3].

In a systematic review conducted by Bellini et al. in 2018, 88 neonates were analyzed, of which only 60 neonates with congenital chylothorax were found to have been treated with octreotide in case series or reports. No prospective or randomized clinical trials were found. Effectiveness was determined by whether there was progressive reduction in chylous drainage, regardless of the time required to reach this response. Octreotide was found to be effective in 53.6% of cases with no difference between preterm and term neonates [4]. It was concluded that octreotide is useful for the treatment of congenital chylothorax regardless of treatment duration. However, there is no consensus regarding dosing regimens. The reported side effects of octreotide range from hyperglycemia, transient cholestasis, transient hypothyroidism, and bloody stools to pulmonary hypertension and necrotizing enterocolitis [3].

We report a neonate with congenital chylothorax successfully treated with continuous administration of octreotide after failure of conservative management.

## 2. Case Report

A premature neonate was born at 33 weeks of gestation via spontaneous vaginal delivery to nonconsanguineous parents with a birth weight of 1.880 kg. She was admitted immediately to the neonatal intensive care unit after initial resuscitation in the delivery room.

Antenatally, there were polyhydramnios, pleural effusion, ascites, and ventricular septal defect. Other than the obvious respiratory distress, initial examination revealed a newborn with dysmorphic features, left-sided cleft lip, and narrow chest.

**Figure 1:**
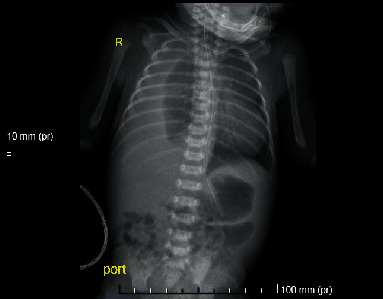
Initial chest X-ray showing right-sided pleural effusion.

Initial chest X-ray revealed an opaque right hemithorax (Figure 1), and it was confirmed to be pleural effusion by chest ultrasound. The patient subsequently required intubation due to the continued respiratory distress. Echocardiography showed ventricular septal defect, secundum atrial septal defect, and small patent ductus arteriosus. Karyotyping for the neonate was normal, screening for congenital infections was negative; however, whole exome genetic sequencing was not done.

**Figure 2:**
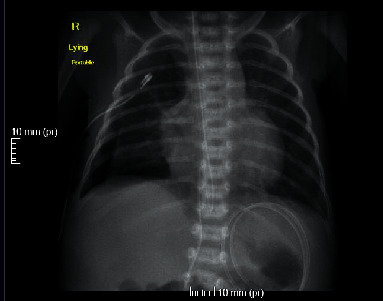
Chest X-ray after insertion of right-sided intercostal tube and octreotide infusion.

Right-sided intercostal tube was inserted, and chylothorax was confirmed by the pleural fluid analysis (Table 1). Conservative management was initiated, and the patient was kept nil per os (NPO) and was started on total parenteral nutrition. Despite these measures, there continued to be high output from the chest tube. A trial of octreotide infusion was initiated at 1 *μ*g/kg/h, and within 24 hours, there was a marked decrease in the amount of chylous output from the chest tube. Initial output was at 100 ml per day prior to the introduction of octreotide, and after four days of continuous infusion, the output declined to 10 ml/day. Detailed management is displayed in Table 2. Chest X-ray was repeated and showed a resolution of the pleural effusion (Figure 2).

## 3. Discussion

The lymphatic system plays a paramount role in maintaining normal physiological functions in humans. Apart from transporting lipids from the enteric circulation to the systemic circulation, it is also important in the regulation of immune factors and the homeostasis of tissue pressures [5]. In neonates, congenital chylothorax can be associated with chromosomal and genetic conditions such as Noonan syndrome, Turner syndrome, and Trisomy 21 or could be due to congenital malformation of the lymphatic system such as lymphangiectasia and cystic hygroma [3].

Chyle is usually an odorless milky fluid, but in neonates who have not started enteral feeding, the fluid obtained via thoracocentesis in congenital chylothorax is serous in nature [6]. In order to establish the diagnosis of chylothorax, a pleural fluid sample should contain a triglyceride level above 110 mg/dl, total cell counts of more than 1,000 cells per mL of fluid with more than 80% lymphocytes [1], and the ratio of cholesterol in the pleural fluid to that in the serum should be less than 1.0 with the presence of chylomicrons [6].

The effects of congenital chylothorax can begin in utero and extend to the postnatal period. Antenatal pleural effusion and pressure effects lead to pulmonary hypoplasia, and protein loss leads to raised interstitial pressure which may lead to hydrops fetalis. After birth, the neonate may suffer from inadequate ventilation and respiratory distress, electrolyte imbalance, hypovolemia, and nutritional depletion and is put at an increased risk of infection due to secondary immunodeficiency [3]. Despite the space occupying lesion effect the congenital chylothorax has on the developing fetus' lung, only 50% of these patients develop symptoms of respiratory distress during the first 24 hours of life and 75% acquire symptoms within the first week [6].

Other than draining the fluid in the pleural space, management aims are to prevent reaccumulation of chylous fluid and to provide nutritional and fluid equilibrium. Treatment modalities vary from medications such as octreotide and sildenafil to surgical procedures or chemical pleurodesis [3].

Octreotide, a synthetic somatostatin analogue, causes mild vasoconstriction in both arterial and lymphatic gut vessels, thus decreasing the hepatic venous flow. It is postulated that this reduction of intestinal absorption may lead to a reduction in chylous flow [4]. It is therefore used as an off-label medication in the management of congenital chylothorax [3].

## 4. Conclusion

Although chylothorax is an uncommon cause of pleural effusion in the pediatric age group, it should be kept in consideration when evaluating a neonate with pleural effusion, as it is the commonest cause of pleural effusion in this age group [6]. No commonly accepted guidelines have been published so far regarding the management of congenital chylothorax in the neonate, but trials of octreotide have appeared to be promising [4]. Further research is needed regarding the incidence of side effects with octreotide, and controlled clinical trials could help establish optimal dosage guides for the duration of treatment and whether continuous infusion versus multiple daily doses are more effective in treating this condition.

## Figures and Tables

**Table 1:** Pleural fluid analysis.

Parameter	Result
Erythrocytes	3000/*μ*L
Leucocytes	1608/*μ*L
Lymphocytes	80%
Monocytes	15%
Plasma cells	0%
Polymorph	2%
Amylase	7 U/I
Chloride	113 mmol/L
Glucose	78 mg/dl
LDH	192 U/I
Total protein	2842.8 mg/dl
Cholesterol	26 mg/dl
Triglycerides	53 mg/dl

**Table 2:** Patient management.

NICU day	Intervention	Accumulated input (ml)	Accumulated output (ml)	Intercostal tube (ml)	Balance (ml)
1	(i) Intubation (ii) Right ICT insertion	—	—	—	—
3	—	77	130	15	−53
4	—	154	239	24	−85
5	—	394	324	30	70
6	—	625	449	30	176
8	—	855	569	50	286
9	—	1111	779	60	332
11	Initiation of total parenteral nutrition (TPN)	—	—	—	—
12	—	1714	1354	100	360
14	Extubated				
15	Octreotide infusion (1 *μ*g/kg/h)	1714	2051	20	−337
16	—	1714	2231	15	−517
17	—	1938	2521	15	−583
19	—	2074	2856	10	−782
20	—	2144	2971	10	−827
23	Removal of right ICT	—	—	—	—
24	(i) Octreotide infusion (0.5 *μ*g/kg/h) (ii) Reintubation after desaturation to 50–60% SpO2	—	—	—	—
27	(i) Octreotide infusion (0.25 *μ*g/kg/h) (ii) Discontinuation of TPN	—	—	—	—
28	Discontinuation of octreotide	—	—	—	—
40	Extubated	—	—	—	—

## Data Availability

The data used to support the findings of this study are available upon request from the corresponding author.
